# Malaria on isolated Melanesian islands prior to the initiation of malaria elimination activities

**DOI:** 10.1186/1475-2875-9-218

**Published:** 2010-07-26

**Affiliations:** 1 Army Malaria Institute, Enoggera, QLD 4051 Australia

## Abstract

**Background:**

The Australian Government's Pacific Malaria Initiative (PacMI) is supporting the National Malaria Program in both Solomon Islands and Vanuatu, complementing assistance from the Global Fund for AIDS, Tuberculosis and Malaria (GFATM). Two remote island groups - Tafea Province, Vanuatu and Temotu Province, Solomon Islands have been selected by the governments of both countries as possible malaria elimination areas. To provide information on the prevalence and distribution of the disease within these island groups, malariometric surveys were conducted during the wet seasons of 2008.

**Methods:**

In Tafea Province, a school-based survey was conducted which included the 2-12 y age group, while in Temotu a village based all-ages survey was conducted. An effort was made to sample villages or schools from a wide an area as possible on all islands. Diagnosis was initially based on Giemsa stained blood slides followed by molecular analysis using polymerase chain reaction (PCR).

**Results:**

In Tafea Province, 73% (5238/7150) of children (2-12 y) were surveyed and in Temotu Province, in the all-ages survey, 50.2% (8742/17410) of the provincial population participated in the survey. In both Vanuatu and Solomon Islands malariometric surveys of their southern-most islands in 2008 showed relatively low over-all malaria parasite prevalence (2 to 3%). Other features of malaria in these island groups were low parasitaemia, low gametocyte carriage rates, low spleen rates, low malaria associated morbidity, a high incidence of asymptomatic infections, and a predominance of *Plasmodium vivax *over *Plasmodium falciparum*.

**Conclusion:**

For various reasons malaria rates are declining in these provinces providing a favourable situation for local malaria elimination. This will be advanced using mass distribution of bed nets and selective indoor residual spraying, the introduction of rapid diagnostic tests and artemisinin combination therapy, and intensive case detection and surveillance. It is as yet uncertain whether malaria parasites can themselves be sustainably eliminated from entire Melanesian islands, where they have previously been endemic. Key issues on the road to malaria elimination will be continued community involvement, improved field diagnostic methods and elimination of residual *P. vivax *parasites from the liver of asymptomatic persons.

## Background

The Melanesian nations of Vanuatu and Solomon Islands have been known for malaria from the reports of the earliest colonial observers; the parasites probably accompanied humans to the archipelagoes many millennia ago [1-4]. Efforts to both quantitate and control malaria through the use of vector control measures such as indoor residual insecticide spraying (IRS) have been difficult due to a change in vector biting behaviour and the isolated nature of the islands with little medical or logistical infrastructure [5-8]. Mosquito surveys indicated that although *Anopheles farauti *was the only vector in Vanuatu, additional vector species constructed a more complex pattern in Solomon Islands [9,10]. Although both nations widely used DDT IRS up until the 1980 s, malaria eradication was not achieved due to technical and financial reasons [5,11]. Reports of parasite drug resistance, especially chloroquine resistance, made therapeutic choices more difficult despite the fact that malaria mortality was low in indigenous populations perhaps due to genetic polymorphisms such as glucose 6 phosphate dehydrogenase deficiency and thalassaemia [12-20]. Early attempts were made to control malaria on the islands using insecticide-treated bed nets (ITN), which eventually became the mainstay of malaria control efforts especially in rural areas [21]. Both *Plasmodium falciparum *and relapsing *Plasmodium vivax *exist in Vanuatu and Solomon Islands usually with a predominance of *P. falciparum*; some observers noted interaction between the species as a possible protective mechanism [15,22,23].

The strong desire of a single island community to have an island free of malaria, primarily for tourism purposes, lead to a series of interventions on Aneityum, the southern-most island of Vanuatu, which included ITN, mass drug administration and mosquito larvae-eating fish [1]. Through a tremendous sustained effort on this island with less than 1000 persons, it was possible to eventually eliminate malaria transmission and maintain it free of re-introduction of parasites for several years. This proof of principle at the natural edge of malaria transmission suggested that shrinkage of the malaria map could occur from Melanesia [24].

**Figure 1:**
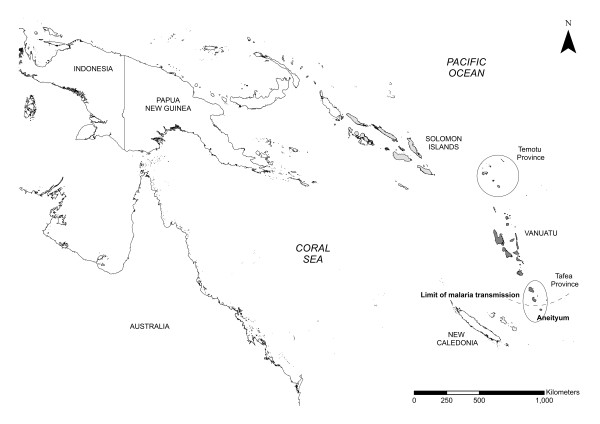
**Map showing survey sites in Vanuatu (Tafea Province) and Solomon Islands (Temotu Province) in relationship to the other countries of the Southwest Pacific region**.

In 2007, the Australian Government initiated through the Department of Foreign Affairs and Trade's external aid organization, AusAID, a programme to support Vanuatu and Solomon Islands in their efforts to control and if feasible eliminate malaria from the islands. Known as the Pacific Malaria Initiative (PacMI), this programme is supported through the Pacific Malaria Initiative Support Centre (PacMISC), a consortium made up of the School of Population Health at the University of Queensland, the Australian Army Malaria Institute and the Queensland Institute of Medical Research. As an entry point to enhanced malaria control possibly leading to elimination, malariometric surveys were conducted in the southern-most provinces of both Vanuatu (Tafea Province) and Solomon Islands (Temotu Province) during 2008 by the National Vector Borne Disease Control Programs in each country with PacMISC support. (See orientation map, Figure 1.) This report contains the results of the two surveys and attempts to extend these point prevalence observations towards what may evolve into a malaria elimination programme for both nations.

## Methods

### Survey region

**Figure 2:**
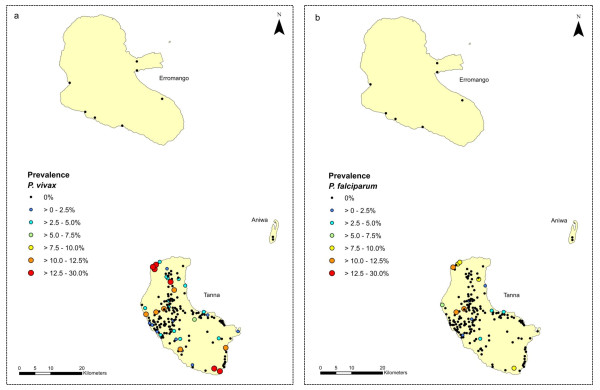
Malaria point prevalence map of Tafea Province, Vanuatu showing parasite rates for a) *P vivax* and b) *P falciparum* based on microscopy results.

Tafea Province lies between 18 - 19°S and 169 - 170°E and is the most southern Province of Vanuatu (Figure 1). The Province is made up of four main islands (Figure 2): Tanna (pop 28,916, including the provincial capital, Lenakel), Erromango (pop 2,062), Aniwa (pop 458), and Aneityum (pop 1,010) from which malaria was eradicated in 1996 [1]. The climate of the Province is tropical monsoon with distinct wet and dry seasons. The median annual rainfall (based on 10 years of data) is 2,400 mm; the wet season, from December to May, accounts for 67% of the annual rainfall; annual mean minimum and maximum temperatures were 20.8°C and 26.4°C respectively. The survey concentrated on Tanna Island, which is the most populated island, although Erromango and Aniwa were also sampled. On the island of Tanna the population is spread over the entire island with the inland plateau region (> 100 m) quite densely populated.

**Figure 3:**
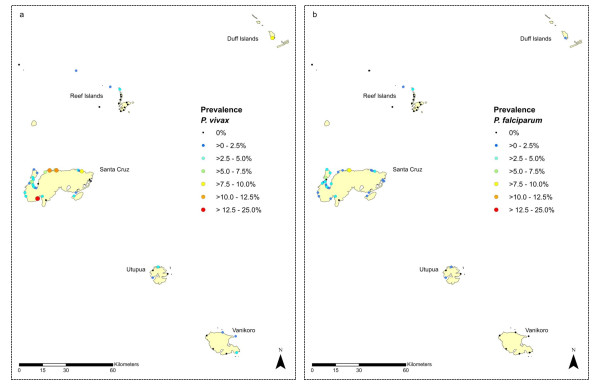
Malaria point prevalence map of Temotu Province, Solomon Islands showing parasite rates for a) *P vivax* and b) *P falciparum* based on microscopy results.

Temotu Province lies between 10 - 12°S and 165 - 167°E, it is the most southern Province in Solomon Islands (Figure 1). The Province is made up of five main island groups (Figure 3): Santa Cruz (or Ndendo; pop 12,112, including the provincial capital, Lata), the Reef Islands (pop 5,958), the Duff Islands (pop 556), Utupua Island (pop 1,300) and Vanikoro island (pop 1,602). The climate is continuous hot/wet with no distinct seasons. The median annual rainfall (based on 38 years of data) is 4,300 mm, each month averages 300-360 mm of precipitation and rainless periods rarely exceeded four days. The annual mean minimum and maximum temperatures are 24.1°C and 30.8°C respectively. All five main island groups were comprehensively covered in the survey. On Santa Cruz, most of the inland plateau region of the island (> 100 m) is uninhabited and nearly all villages are located along the coast.

### Field work and ethics approvals

A school-based, mass blood survey of children was conducted in Tafea Province, Vanuatu and a village-based mass blood survey of all ages was conducted in Temotu Province, Solomon Islands. With accurate lists of schools and villages, a sampling plan was designed to comprehensively cover each island. Schools and villages were visited one - two days prior to the survey to confirm approval and support. On the day of the survey, the purpose and procedures of the survey were explained to participants at each location. Blood was collected by finger stick by personnel from the respective Vector Borne Disease Control Programmes with explicit community and oral individual consent, which in the case of children was given by the parent, guardian or carer (teacher) [25,26]. The blood collected was used for a thick and thin blood film for microscopic diagnosis and for a blood spot (20-30 μl) dried on filter paper (Whatman No.3) for molecular diagnosis, the blood spot was preserved desiccated on silica gel until processed. Basic demographic and malaria history information was solicited from each participant by the Vector Borne Disease Control Programme staff. Tympanic temperatures were taken and all febrile (> 38.0°C) subjects immediately tested for malaria using a rapid diagnostic test (RDT) (ICT Malaria, ICT Diagnostics); all positive cases were treated as per the respective national treatment guidelines. All blood slides were read within 48 hours and any positive cases were followed up with treatment given, though full compliance could not always be observed and no follow up studies were done to document cure. In three villages on Santa Cruz, spleens were palpated on all persons participating in the study.

Approval for the malaria surveys were obtained from the Vanuatu Medical Ethics Committee (dated 13 Oct 2008) and Solomon Islands Health Research Ethics Committee (dated 12 Sep 2008). Ethical approval for obtaining filter paper blood spots from the same finger stick used to prepare malaria blood smears was obtained from the Australian Defence Force Human Research Ethics Committee (ADHREC 507/07) in order to store the filter paper blood spots and test them for malaria afterwards once they had been made anonymous by removal of personal identifiers.

### Diagnosis by microscopy

Thick and thin blood smears were prepared. The thin film was fixed in methanol and the slide stained with 10% Giemsa for 15 min. To determine positivity 100 thick film fields were examined under oil-immersion at 1000×. If positive, the slide was reread against 200 - 500 white blood cells depending on the parasite density. Malaria microscopists who participated in these surveys were all certified to WHO malaria microscopy standards by prior participation in WHO accredited malaria microscopy competency assessment in Brisbane or Honiara. Microscopists who performed the initial slide examinations were all certified to competency levels 1 (expert), 2, or 3. Microscopists who performed the quality assurance were certified to level 1 or 2 and all slide examinations requiring a tie-breaking "referee" reading were certified to level 1. All slides, which were considered positive on the initial examination, all microscopy initial examination negative/PCR positives samples, and a 10% sample of microscopy initial examination negative samples, were subjected to a second quality assurance (QA) examination. All slides which subsequently recorded discrepant results between the initial examination and the QA examination were then subjected to a third "referee" examination, which was recorded as the final microscopy result.

### Diagnosis by molecular analysis

All blood spots from the Tafea survey, and a subset of blood spots from the Temotu survey which include all febrile, microscopy positive samples and 10% randomly selected from different locations, were processed at the Australian Army Malaria Institute, Brisbane, Australia. DNA was extracted using QIAGEN QIAamp DNA Mini Kits and a QIAcube robot (QIAGEN, Crawley, U.K.). The manufacturer's protocol (QIAamp DNA Mini and Blood Mini Handbook 2E) was followed, except only one 5 mm circle was extracted per sample, and eluted to a 100 μl volume. Initial analysis was by multiplexed PCR using previously published primers [27]. Following this, all putative positive samples was subjected to a second round of testing, where parallel reactions identical to that described above but using only one forward primer per reaction allowed more definite speciation. A smaller, randomized sample was analysed in the same way, for quality assurance purposes.

### Mapping

Though the survey in Tafea Province was a school-based survey additional information was collected on the village in which the child would normally sleep. These schools and villages were later geo-referenced using data provided by the respective National Ministry of Lands and 1:50,000 scale topographic maps of the islands. The survey in Temotu Province was village-based with coordinates of each village obtained at the time of the survey using GPS (Trimble, Juno™ST Handheld). Based on the results from the slide microscopy, *P. falciparum *and *P. vivax *prevalence in each village where the person slept the night previously and surveyed village for Tafea and Temotu provinces respectively from where the blood was sampled were mapped using the GIS software ArcView version 9.3 (ESRI, Redlands, CA).

## Results

### Geographic point prevalence

Figure 1 shows the position of the provinces which participated in the malaria surveys within the Southwest Pacific region. Figure 2 (Tafea Province, Vanuatu) and Figure 3 (Temotu Province, Solomon Islands) show the geographic point prevalence of malaria, both falciparum and vivax, as found during 2008 by the methods described above. The concentration of malaria parasitaemia on the main island of each province and relative paucity of parasites on the outer islands is a feature of both island groups. Although some children with parasitaemia where found in the interior of Tanna, entomological and historical information lead us to think that many if not most of these infections were obtained while visiting the sea coast. The concentration of population in coastal villages in all the other islands besides Tanna also accounts for the geographic concentration of parasitaemia along the coast.

### Parasite species composition

*Plasmodium falciparum *and *P. vivax *were the only two species found in Tafea and Temotu Provinces. In both Provinces *P. vivax *was the dominant species accounting for 69.4% of infections in Tafea and 62.7% in Temotu. Mixed infections occurred in 7.6% and 11.9% of infections in Tafea and Temotu Provinces respectively.

### Parasite prevalence within and between islands

In Tafea Province 73% (5238/7150) of children (2-12 y) were surveyed; in Temotu Province, in the all-ages survey, 50.2% (8742/17410) of the provincial population was included in the survey. The point prevalence malaria rate is shown in Table 1 (Tafea Province, Vanuatu) and Table 2 (Temotu Province, Solomon Islands) with Table 3 adapted for comparison between the two Provinces different age groups. Both Tafea and Temotu Provinces consist of multiple islands some of them quite small and isolated, however > 70% of the provincial population was found on a single island. Although larger than Tanna in area, Erromango has < 10% of Tanna's population. No positive blood smears were found on Erromango out of 413 children tested although 2 were positive by PCR. No positive findings were obtained from Aniwa. Temotu Province consists of five island groups whose all-species parasite prevalence varies greatly: Santa Cruz (Ndendo island) (4.3%), Reef Islands (1.5%), Duff Islands (11.6%), Utupua (1.9%) and Vanikoro (1.8%).

**Table 1:** Malaria parasite species and prevalence detected by microscopic and molecular diagnosis in Tafea Province, Vanuatu 2008.

		Number Positive (%)		
		
Age group	Number	Pf	Pv	Pf/Pv
0-2 y	369	3	13	0
3-5 y	1269	7	17	4
6-8 y	1897	15	32	4
9-11 y	1700	16	31	3
12 y	3	0	0	0

Total	5238	41 (0.8)	93 (1.7)	11 (0.2)

Pf = *P. falciparum*; Pv = *P. vivax*; Pf/Pv = mixed infection

**Table 2:** Malaria parasites species and prevalence determination by microscopic and molecular diagnosis in Temotu Province, Solomon Islands 2008.

		Number Positive (%)		
		
Age group	Number	Pf	Pv	Pf/Pv
0-4 y	1533	16	35	6
5-9 y	1696	26	57	12
10-14 y	1261	17	33	10
15-29 y	1832	26	41	6
30-44 y	1324	19	16	2
45-59 y	736	4	4	1
60+	360	3	1	0

Total	8742	111 (1.3)	187 (2.1)	37 (0.4)

Pf = *P. falciparum*; Pv = *P. vivax*; Pf/Pv = mixed infection

**Table 3:** Percent parasite prevalence by age group for Tafea Provence, Vanuatu and Temotu Province, Solomon Islands 2008.

Age group	Tafea Province	Temotu Province
0-4	2.52%	3.72%
5-9	1.78%	5.60%
10-14	2.33%	4.76%
15-29	-	3.98%
30-44	-	2.79%
45-59	-	1.22%
60+	-	1.11%

Considerable heterogeneity in parasite rates was seen across the larger islands of Tanna and Santa Cruz. In some schools and villages low or no malaria prevalence was found, while schools and villages less than a 1-2 km away, and within the flight range of the vector, had parasite rates of > 7.5% (Figures 2 and 3). A similar heterogeneity was also seen with the species composition; the predominance of *P. vivax *was by no means uniform across these islands, and *P. falciparum *was the dominant species in a number of locations (Figure 2 and 3).

### Parasitaemia

In the all age survey conducted in Temotu Province the density of asexual forms were low for all infections. In 320 infections, 61% had < 100 parasites/μl, 81.0% had < 500 parasites/μl, 89.3% had < 1,000 parasites/μl, and 10.7% had > 1,000 parasites/μl.

Gametocyte carriage rates based on the total number of slides taken were 0.26% for *P. falciparum *(15.1% of *P. falciparum *positives) and 0.09% for *P. vivax *(4.3% of *P. vivax *positives)

### Spleen rates

In Temotu Province. from three villages 331 spleens were palpated from all age groups. Using the Hackett classification system, three were Class 1, four were Class 2, and one was Class 3, giving a spleen rate of 2.4% and an average enlarged spleen index of 0.04. Of the eight enlarged spleens six were in children (0-7 y).

### Parasite prevalence by age and gender

For Temotu there was no statistically significant difference (p > 0.1) between the number of infections found in each age group (Table 3); implying that there is little age related exposure and thus little acquired immunity. Few gender differences were noted during the surveys in terms of malaria parasite prevalence. In Tafea Province, Vanuatu 52% of all those surveyed were male whereas 59% of parasite positive children were male. In Temotu Province, Solomon Islands 47% of all participants were male whereas 51% of those with parasitaemia were male.

### Lack of symptoms particularly fever

In Tafea Province, only 28% of children that were slide positive were febrile (> 37.5°C) (O.R.; 95% C.I. = 1.7; 1.1 to 2.6) although 19% of all slide negative subjects had an aural temperature > 37.5°C. Among all ages tested in Temotu Province, Solomon Islands, only 14% of those with confirmed parasitaemia, were febrile (2.1; 1.6 to 2.9), although overall, 7% of those participating in the survey had an aural temperature > 37.5°C. People in Temotu with falciparum malaria detected by blood smear were more likely to have a fever > 38°C than those with vivax malaria (3.2; 1.1 to 9.7). Despite the significant association of fever with malaria parasitaemia, this was of limited usefulness due to the low parasitaemia rate. For example, passive detection by taking blood smears only on those with a temperature > 37.5°C would not be very effective as the positive predictive value (true positive/(true positive +false positive) for a febrile child in Tafea having falciparum parasitaemia detected by microscopy was < 1% and for any parasitaemia detected by PCR was 4%. Similarly in all ages in Temotu, a temperature > 37.5°C had a positive predictive value of for falciparum parasitaemia detected by microscopy of 1.69% and fever had a positive predictive value of any parasites being found by PCR of 7%.

## Discussion

The malaria surveys reported here were conducted to develop a detailed cross-sectional dataset from the southern-most islands of two nations in order to inform a revised action plans for newly resourced national malaria programmes in Solomon Islands and Vanuatu [25,26]. These surveys and past historical data characterize the epidemiology of malaria in these isolated island groups as hypoendemic having: low prevalence rates, low parasitaemia, low morbidity with a high incidence of asymptomatic cases, low spleen rates, low gametocyte carriage rates, presumably low immunity within the population, an absence of epidemics and a predominance of *P. vivax*. These parameters are atypical of those previously found in other provinces of both Vanuatu and Solomon Islands [28-31], where transmission is more intense with higher parasite rates (up to 35%), higher gametocyte carriage rates, higher spleen rates, and a predominance of *P. falciparum*. However two features that were comparable with other parts of Vanuatu and Solomon Islands and also in Melanesian Papua New Guinea was low morbidity with a high incidence of asymptomatic infections and low parasitaemia [30-32]. In both Tafea and Temotu Provinces the only anopheline and thus the only vector was *Anopheles farauti*. This is primarily a coastal species capable of breeding in brackish water; it is a competent malaria vector throughout its range which includes all the Melanesian islands of Papua New Guinea, Solomon Islands and Vanuatu [28,33,34].

Both Provinces were known from routine health service information to have a relatively small malaria problem and this was confirmed by these surveys. Because of the low malaria burden and isolation from population centers, it was felt that both Provinces might be candidates for malaria elimination in the near future. The differing percentages of parasitaemia and species within and between island groups highlight the importance of sampling from as wide an area as possible in order to better target specific approaches to elimination. At a minimum the surveys were designed to give a better idea of what might be possible given enough staff and materials to obtain very high ITN coverage and improved case management. A malaria survey can act not only as a data collection exercise but also as a programmatic tool to concentrate minds and reinvigorate staff with new ideas and possibilities. Surveys can also be a good means to determine the level of community interest and cooperation, both of which are critical to any enhanced public health program regardless of its specific goal.

The mass blood surveys reported here are probably the most comprehensive ever conducted in these provinces. There are multiple reasons why parasite prevalence might show the heterogeneity seen here both within and between islands. The availability of suitable larval habitats for the vector and the location of villages with respect to these larval habitats; the movement and transit of infected persons (particularly those harbouring hypnozoites), this movement may be limited between islands, but is probably very extensive within islands as extended families link communities and villages; also access to preventive measures such as ITN or effective chemotherapy will impact on parasite rates. While spatially expansive, surveys of this type are temporally limited and may not indicate fluctuations in transmission rates that could occur at other times of the year or in different seasons. Malariometric surveys previously conducted on the island of Espiritu Santo in Vanuatu indicated that parasite rates varied between wet and dry seasons as did species composition with *P. falciparum *dominant in the wet season and *P. vivax *dominant in the dry season [23]. The present surveys in Tafea and Temotu Provinces reported here were conducted towards the end of the wet season when transmission would be at its peak. Despite this the overall malaria prevalence was only 2-3% with a predominance of vivax malaria. This follows a trend, which commenced in the early 1990 s, towards lower transmission rates for these remote island groups, and for the other Provinces in both Solomon Islands and Vanuatu as well [2,5,6,30]. Despite the development of chloroquine resistance in *P. falciparum *and in to a lesser degree *P. vivax *during the 1980 s [18,19], the downward trend in malaria has been driven by a general improvement in the overall health of the community, promotion of a greater awareness of malaria, and the efforts of the respective national Vector Borne Disease Control Programs through better access to treatment and vector control measures [30,35]. One can use the proportion of vivax malaria as a rough gauge of malaria elimination suitability as malaria control activities preferentially stop falciparum malaria transmission leaving residual liver parasites of *Plasmodium vivax *to relapse and re-infect mosquitoes months to years later. After malaria transmission is stopped, incident vivax malaria cases will more likely be due to relapses from liver parasites and not recrudescences from asymptomatic bloodstream infections.

Remarkably little morbidity and no mortality could be ascribed to malaria during the surveys. This has been previously described in other parts of Melanesia where it has been attributed to a progressive acquisition of anti-parasitic immunity [30,32]. However, in Tafea and Temotu where transmission rates are very low, exposure would seem to be insufficient to allow immunity to be maintained as indicated by the few age-related differences in parasitaemia. Other hypotheses presented to explain this observation include interactions between different malaria species or widely distributed human genetic polymorphisms (e.g. thalassaemia); both these hypothesis, however, seem at best incomplete [12,20,22,36]. If malaria is rapidly being removed as a major public health problem in these areas, will there be sufficient community interest to maintain programmes to eliminate malaria? Such a loss of community support in previous Malaria Eradication and Control programs using DDT IRS was indicated by a refusal to allow houses to be sprayed as malaria was no longer perceived as a health problem.

For elimination to ultimately succeed, there must be an effective barrier to reintroduction of the parasite. This is a major concern in the malarious countries of Africa, Asia and South America where international boarders are porous and intra-country cooperation will be critical in maintaining malaria free areas [24]. One advantage in attempting elimination in remote island groups, such as Tafea and Temotu, is that their very isolation will help reduce the chances of reinvasion. Travel to Tafea and Temotu is limited to a light aircraft service and one or two cargo barges per month; there is no other way to access these Provinces. On Aneityum all incoming passengers (two flights per week) have a blood slide taken and read by a community microscopist; this appears to have been sufficient in monitoring and preventing a reintroduction of the parasite [1]. The major difference between a malaria programme aimed at sustained control and one intending to eliminate parasite transmission is the intensity of surveillance required. Low morbidity and a high incidence of asymptomatic infections weaken the effectiveness of passive and active case detection as a tool for monitoring and evaluating elimination programs. With regards to mass blood surveys, although a community may be happy to submit to finger sticks on an occasional basis, this might not be true if frequent blood slides are needed in focal areas to locate and eliminate the few remaining parasites.

Additionally these types of surveys are financially and logistically demanding. It may be necessary to think of other disease control activities that can be done within the epidemiological framework of a malaria elimination campaign in order to positively reinforce community participation. For example, although yaws treatment has nothing specifically to do with malaria elimination, treatment of such a visible neglected health problem may be critical to maintaining community involvement with malaria interventions.

The implications of these survey data for malaria elimination activities are several. Low and decreasing parasitaemia prevalence in isolated island communities indicate that a concerted push might be able to interrupt malaria transmission. Some of the many conditions that apply to such efforts would include having effective interventions acceptable to the community as well as means to find the last remaining parasitaemic individuals and then detect any re-introductions before transmission can be re-established. The single greatest current need for malaria elimination programmes may be a simple, highly-sensitive, easy-to-use, affordable diagnostic test whose use would allow the rapid screening of large numbers of people looking for very few parasitaemic individuals. Further research and development in this regard are urgently required as microscopic blood smears are very difficult to do if the positivity rate is < 1:1,000 and PCR is slow and expensive. The ability to kill the last parasite and thus eliminate malaria will critically depend on being able to locate the person within whom the last parasite is hiding.

## Conflict of interests

No member of the study group reports any conflict of interest. Their respective governmental organization membership is shown under member's contributions.

## Authors' contributions

GDS and RC contributed the design of the studies and wrote the manuscript.

AB, IR, GT, AV and MW designed the studies, developed the protocols, obtained various ethics approvals, interpreted the data, and contributed to the preparation of the manuscript. AA, BA CA JAA, LB, LMB, AC, QC, ED, GD, AE, MDE, NE, SF, KAG, BH, IH, IEH, JH, SI, JI, MLJ, DK, GK, MJK, BL, KL, SL, BM, DM, LM, KM, NM, SM, AN, LO, RP, HR, NR, RR, JS, WWS, AT, NW and JY contributed to the collection and processing of the samples, quality assurance of results, data collation and analysis, and contributed to the preparation of the manuscript. All the authors have read and approved the final manuscript.
